# Bile acid production is life-stage and sex dependent and affected by primer pheromones in the sea lamprey

**DOI:** 10.1242/jeb.229476

**Published:** 2021-05-10

**Authors:** Yu-Wen Chung-Davidson, Ugo Bussy, Skye D. Fissette, Anne M. Scott, Weiming Li

**Affiliations:** Department of Fisheries and Wildlife, Michigan State University, Natural Resources Building, Rm. 13, 480 Wilson Road, East Lansing, MI 48824, USA

**Keywords:** Petromyzonamine, Petromyzonene, Petromyzonol, Petromyzonsterol, *Petromyzon marinus*

## Abstract

Pheromonal bile salts are important for sea lampreys (*Petromyzon marinus*) to complete their life cycle. The synthesis and release of a releaser/primer pheromone 3-keto petromyzonol sulfate (3kPZS) by spermiating males have been well characterized. 3kPZS evokes sexual behaviors in ovulatory females, induces immediate 3kPZS release in spermiating males, and elicits neuroendocrine responses in prespawning adults. Another primer pheromone released by spermiating males, 3-keto allocholic acid (3kACA), antagonizes the neuroendocrine effects of 3kPZS in prespermiating males. However, the effects of 3kACA and 3kPZS on pheromone production in prespawning adults is unclear. To understand the foundation of pheromone production, we examined sea lamprey bile salt levels at different life stages. To investigate the priming effects of 3kACA and 3kPZS, we exposed prespawning adults to vehicle or synthetic 3kACA or 3kPZS. We hypothesized that endogenous bile salt levels are life-stage and sex dependent, and differentially affected by 3kACA and 3kPZS in prespawning adults. Using ultra-performance liquid chromatography tandem mass spectrometry, we found that sea lampreys have distinct mixtures of bile salts in the liver and plasma at different life stages. Males usually had higher amounts of bile salts than females. Petromyzonamine disulfate was the most abundant C_27_ bile salt and petromyzonol sulfate was the most abundant C_24_ bile salt. Waterborne 3kACA and 3kPZS exerted differential effects on bile salt production in the liver and gill, their circulation and clearance in the plasma, and their release into water. We conclude that bile salt levels are life-stage and sex dependent and differentially affected by primer pheromones.

## Abstract (precis)

**Summary:** Sea lamprey bile salts are pheromones produced in a sex- and life-stage-dependent manner. Primer pheromones differentially affect bile salt production, circulation, clearance and release.

## INTRODUCTION

The sea lamprey (*Petromyzon marinus*) is a vertebrate residing at an important evolutionary juncture where jawed vertebrates (Gnathostomata) branch out from jawless vertebrates (Agnatha). Chemical communication is important for sea lampreys to complete their life cycle, including the prespawning migration aided by migratory pheromones released from larvae (Bjerselius et al., 2000; Dvornikovs et al., 2006; Hoye et al., 2007; Li et al., 2018a; Sorensen et al., 2005), and reproduction facilitated by sex pheromones released from spermiating males (Buchinger et al., 2017; Chung-Davidson et al., 2013a; Li et al., 2002; Scott et al., 2019; Siefkes et al., 2005). The pheromonal bile salt 3-keto petromyzonol sulfate (3kPZS) released by spermiating males induces sex-dependent reproductive behaviors in spawning adults (Buchinger et al., 2013, 2017; Li et al., 2002; Siefkes et al., 2005), and increases immediate 3kPZS release and hepatic production in spermiating males upon detection (Fissette et al., 2020). However, another primer pheromone released by spermiating males, 3-keto allocholic acid (3kACA), seems to antagonize the neuroendocrine effects of 3kPZS in prespermiating males. Waterborne 3kACA and 3kPZS act on different olfactory receptors (Siefkes and Li, 2004) and differentially prime the neuroendocrine system in prespawning adults (Chung-Davidson et al., 2013a,b, 2020a,b). However, their priming effects on bile salt production in prespawning adults are still unknown.

**Fig. 1.**
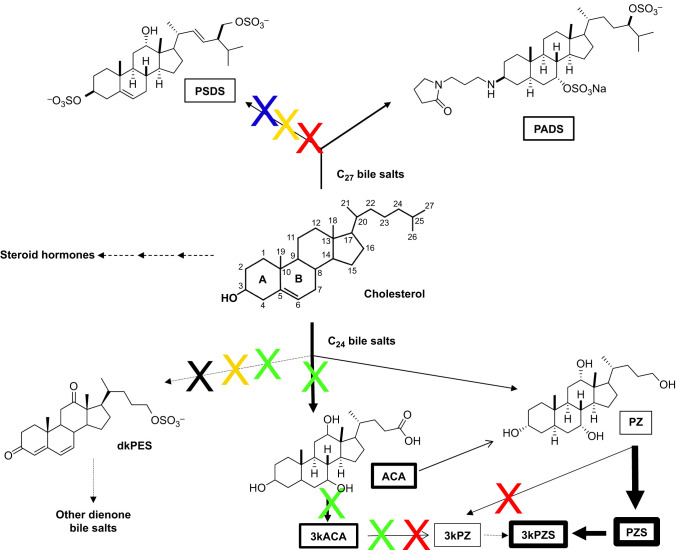
**Sea lampreys produced distinct mixtures of bile salts at different life stages.** Lamprey-specific bile salts are derivatives of cholesterol, with most modifications at C3, C5, C7, C12 and C24 positions. Cholesterol can be converted to trace amounts of steroid hormones such as androstenedione, estradiol, progesterone, testosterone and 15α-hydroxyprogesterone in sea lampreys, but the major steroid products are bile salts. Depending on the occurrence of side-chain cleavage, cholesterol can be converted into 27-carbon (C_27_) or 24-carbon (C_24_) bile salts. Larval sea lampreys do not produce 3,12-diketo-4,6-petromyzonene-24-sulfate (dkPES), whereas spermiating males do not produce petromyzonsterol disulfate (PSDS). Preovulatory females produce neither PSDS nor 3-keto petromyzonol (3kPZ), whereas prespermiating males produce neither PSDS nor dkPES. Ovulatory females do not produce allocholic acid (ACA), 3-keto allocholic acid (3kACA), dkPES or 3kPZ. The relative abundance of bile salts in larval liver is indicated by the line thickness of the arrows and enclosed rectangles, based on results from this study. Dashed lines indicate trace or no detectable conversions. Black cross: conversion not detected in larvae; green cross: conversion not detected in ovulatory females; blue cross: conversion not detected in spermiating males; red cross: conversion not detected in preovulatory females; yellow cross: conversion not detected in prespermiating males. PADS, petromyzonamine disulfate; PZ, petromyzonol; PZS, petromyzonol sulfate (structure not shown); 3kPZS, 3 keto-petromyzonol sulfate (structure not shown). A and B indicate the A ring and B ring in the cholesterol structure, respectively.

Bile salts are the prominent steroids in sea lampreys and are derivatives of cholesterol as in other vertebrates (Elliott and Hyde, 1971; Hagey et al., 2010; Russell and Setchell, 1992; Salen and Shefer, 1983). In addition to cholic acid, taurocholic acid and taurodeoxycholic acid that are commonly found in other vertebrates, sea lampreys also produce higher amounts of lamprey-specific bile salts (Wang et al., 2015). Depending on the sequence of enzymatic modifications and the occurrence of side chain cleavage, cholesterol can be converted into trace amounts of steroid hormones (Chung-Davidson et al., 2020b) or lamprey-specific bile salts with either a 27-carbon (C_27_) or 24-carbon (C_24_) backbone (Fig. 1) (Hagey et al., 2010; Haslewood and Tökes, 1969; Li et al., 2002, 2017a, 2018c; Yun et al., 2003). The diversity of sea lamprey bile salts mostly come from the saturation status of the carbon bond at the A/B ring junction, the substitution of functional groups (carboxyl/hydroxyl/keto/sulfate), and the conjugations at 3, 7, 12 and 24 carbon positions (Li et al., 2018b). Known sea lamprey bile salts can be classified into three groups including: (1) C_27_ bile salts: petromyzonamine disulfate (PADS) and petromyzonsterol disulfate (PSDS); (2) C_24_ bile salts with multiple double bonds: 3,12-diketo-4,6-petromyzonene-24-sulfate (dkPES) and several bile salt-like dienones; and (3) a group of structurally related C_24_ bile salts: 3kPZS and its presumptive precursors petromyzonol sulfate (PZS), petromyzonol (PZ), 3-keto petromyzonol (3kPZ), allocholic acid (ACA) and 3kACA (Fig. 1) (Buchinger et al., 2015; Hoye et al., 2007; Li et al., 2002, 2012, 2013, 2017b, 2018c; Yun et al., 2003).

Interestingly, pheromones emitted by one individual can affect the rates of its production in other individuals. Honeybee queen pheromones can inhibit workers from producing queen-like substances and promote worker sterility and worker-like pheromone composition. In the absence of queen pheromones, workers become fertile and express the queen pheromones (Tamar et al., 2006). In sea lampreys, similar inhibition induced by pheromonal bile salts in prespawning adults has yet to be investigated. As 3kACA and 3kPZS act on different olfactory receptors (Siefkes and Li, 2004) and exert differential neuroendocrine responses (Chung-Davidson et al., 2013a,b, 2020a,b), we hypothesize that bile salt levels are life-stage and sex dependent and differentially affected by 3kACA and 3kPZS in prespawning sea lampreys. Using ultra-performance liquid chromatography tandem mass spectrometry (UPLC-MS/MS; Li et al., 2011; Wang et al., 2013, 2015), we found life-stage and sex differences in bile salt levels in sea lampreys, and 3kACA and 3kPZS differentially altered bile salt levels in prespawning adults and spermiating males. Waterborne 3kPZS also increased PZS, 3kPZS, ACA and 3kACA water release in spermiating males.

## MATERIALS AND METHODS

### Animals

Prespawning migratory *Petromyzon marinus* Linnaeus (mean±s.e.m. body length 46.8±0.3 cm, body mass 212.3±3.8 g) were collected from Saint Marys River in 2014 and 2015 by agents of the US Fish and Wildlife Service (Marquette, MI, USA) and Department of Fisheries and Oceans Canada Sea Lamprey Control Centre (Sault Ste Marie, ON, Canada). Larval sea lampreys (body length 11.0±0.4 cm, body mass 2.0±0.2 g) were collected by the survey crew of US Fish and Wildlife Service, Ludington Biological Station (Ludington, MI, USA) in 2016. They were sent to US Geological Survey, Hammond Bay Biological Station, Great Lakes Science Center (Millersburg, MI, USA). Prespermiating males were visually identified and separated from preovulatory females by carefully applying pressure to the lower abdomen to feel for eggs. Ovulatory females and spermiating males were obtained by holding prespawning animals in cages (0.25–1.0 m^3^) in the lower Ocqueoc River (at US23 bridge, Millersburg, MI, USA) to induce maturation (Brant et al., 2013). They were checked daily for gamete release by applying gentle pressure to the lower abdomen. All animals were then transferred to the University Research Containment Facility at Michigan State University where pheromone treatments and sample collections were performed in early August in 2014 and 2015. Life-stage sample collections were performed in summer of 2015 and 2016. Standard operating procedures for transporting, maintaining, handling, anesthetizing and euthanizing sea lampreys were approved by the Institutional Committee on Animal Use and Care of Michigan State University and in compliance with standards defined by the National Institutes of Health Guide for the Care and Use of Laboratory Animals (https://www.ncbi.nlm.nih.gov/books/NBK54050/pdf/Bookshelf_NBK54050.pdf).

### Chemicals

Acetonitrile (ACN), ethanol, methanol and water were HPLC grade (Mallinckrodt Baker Inc., Phillipsburg, NJ, USA). Ammonium acetate (HPLC grade) and ethyl 3-aminobenzoate (MS222) were purchased from Sigma-Aldrich Co. (St Louis, MO, USA). PZ and 3kPZ were purchased from Cayman Chemical (Ann Arbor, MI, USA). ACA, 3kACA, dkPES, PZS, 3kPZS, PADS, PSDS and deuterated [^2^H_5_]3kPZS were custom-synthesized from Bridge Organic Inc. (Vicksburg, MI, USA).

### Pheromone treatments

Prespawning sea lampreys were acclimated for 2 days in a 200 l tank with a water temperature of 16±1°C and outflow rate of 1 l min^−1^. Animals (6 per tank) were randomly assigned to treatment groups (0.05 ppm methanol vehicle, 10^−10^ mol l^−1^ 3kACA or 10^−10^ mol l^−1^ 3kPZS) and treated for 4 h. The dosage and exposure time were chosen according to previous results (Chung-Davidson et al., 2013a,b). During the treatment period, water was aerated but not replenished. Water samples (10 ml) were collected from each tank immediately before and after the treatment (repeated measures). Animals were anesthetized with 0.02% MS222 after treatment. Blood samples were collected via cardiac puncture with 10 ml heparinized vacutainers and kept on ice before processing for plasma (supernatant) by centrifugation at 1000 ***g***, 4°C for 20 min. Animals were decapitated and liver and gill samples were snap-frozen in liquid nitrogen and stored at −80°C until processing for bile salt UPLC/MS-MS quantification.

### Life-stage sample collection

To measure bile salt concentration in sea lampreys at different life stages, plasma samples were collected from 40 preovulatory females, 5 ovulatory females, 43 prespermiating males and 59 spermiating males. Ovulatory females were hard to obtain because they died soon after spawning. Because of the difficulty in obtaining larval blood (1–2 µl per larva), we did not collect larval plasma samples. Whole liver from 21 larvae (with the embedded gall bladder), and small pieces of liver tissue from 24 preovulatory females, 5 ovulatory females, 23 prespermiating males and 23 spermiating males were collected to determine bile salt production in the liver. Sample size varied according to availability at the time.

### UPLC-MS/MS quantification of bile salts

Tissue samples were weighed (larval liver: 32.2±1.6 mg, adult liver: 308±8 mg, adult gill: 431±51 mg) and homogenized with 400 μl of 75% ethanol (in HPLC water) with 1 ng internal standards (100 ng ml^−1^ [^2^H_5_]3kPZS; 10 µl per sample). The final volume of the liver homogenate was brought up to 1.5 ml by adding 1100 μl of 75% ethanol. To dissolve and extract bile salts, liver homogenate (in 75% ethanol) was incubated at room temperature overnight, and centrifuged twice at 15,800 ***g***, 4°C for 20 min. The supernatant was transferred to a new tube and freeze-dried using a CentriVap Cold Trap Concentrator (Labconco Co., Kansas City, MO, USA.). Plasma samples (100–500 µl, depending on availability) were processed similarly but without homogenization. Water samples were freeze-dried directly. Samples were then reconstituted with 1 ml 50% methanol (in HPLC water) and transferred to autosampler vials before UPLC-MS/MS analyses.

Solutions for the calibration standard curve and the internal standard were prepared freshly from 1 mg ml^−1^ stock solutions of respective compounds in 50% methanol. All stock solutions were stored at −20°C until use. For each UPLC-MS/MS analysis, working solutions were prepared daily from the stock solution, and analyzed by full scan MS to ensure that no detectable contamination or degradation occurred. To prepare the working solutions of the calibration standard curve, appropriate amounts of the respective standard compound stock solution were serially diluted to produce a calibration standard curve from 0.01 to 1000 ng ml^−1^. A Waters (Milford, MA, USA) Xevo TQ-S mass spectrometer coupled with a Waters ACQUITY H-Class UPLC system was used. A Waters BEH C18 column (2.1×50 mm, 1.7 µm particle size) was used with the column oven temperature set at 30°C. Mobile phase A was 10 mmol l^–1^ ammonium acetate in water, and mobile phase B was ACN. Separation was achieved using the following gradient program at a flow rate of 200 l min^−1^ for 15 min: 3% B for 0.5 min, increased to 40% B from 0.5 to 10 min, increased to 95% B from 10 to 12 min, maintained at 95% B from 12 to 13 min, and returned to 3% B from 13 to 15 min. The injection volume was 10 µl. Mass spectra were acquired using electrospray ionization in negative ion mode with multiple reaction monitoring. The capillary voltage, extractor voltage and rf lens settings were 3.17 kV, 4 V and 0.3, respectively. The flow rates of cone gas and desolvation gas were 20 and 400 l h^−1^, respectively. The source temperature and desolvation temperature were set at 110 and 350°C, respectively. Collision-induced dissociation employed argon as the collision gas at a manifold pressure of 2×10^−3^ mbar (200 Pa), and collision energy and source cone potentials were optimized for each transition using Waters QuanOptimize software. Data were acquired with MassLynx 4.1, and calibrated and quantified by QuanLynx software.

Extraction efficiency (recovery) for sea lamprey bile salts ranged from 76.7±6.2% to 85.9±7.0% in gill homogenates; 76.3±5.2% to 98.7±8.6% in liver homogenates; 70.5±5.5% to 89.1±6.7% in plasma; and 84.1±3.2% to 90.3±2.2% in water (Li et al., 2011; Wang et al., 2013, 2015). The limit of detection for lamprey bile salts ranged from 0.009 ng ml^−1^ to 0.02 ng ml^−1^ in plasma and tissues, and 0.4 pg ml^−1^ to 0.05 ng ml^−1^ in water (Li et al., 2011; Wang et al., 2013, 2015).

### Statistics

One-way analysis of variance (ANOVA) was used to compare the bile salt concentration in tissues and plasma (normalized by mass or volume) among different life stages or different pheromone treatment groups. Bonferroni/Dunn *post hoc* tests (Dunn multiple comparison *post hoc* tests with Bonferroni correction) were performed if the ANOVA test was significant (*P*<0.05). Some prespermiating males began spermiating at the time of sample collection, and these data were separated as groups of spermiating males. Liver sample size: *n*=6 per group except prespermiating males in the control (*n*=11), 3kACA-treated (*n*=12) or 3kPZS-treated (*n*=9) groups, and spermiating males in the 3kACA-treated group (*n*=3). Plasma sample size: preovulatory females in the control group *n*=8, 3kACA-treated group *n*=8 and 3kPZS-treated group *n*=10; prespermiating males in the control group *n*=13, 3kACA-treated group *n*=12 and 3kPZS-treated group *n*=7; spermiating males in the control group *n*=23, 3kACA-treated group *n*=16 and 3kPZS-treated group *n*=26. Gill sample size: *n*=6 per group except prespermiating males in the 3kPZS-treated group (*n*=3), and spermiating males in the control (*n*=11), 3kACA-treated (*n*=9) and 3kPZS-treated (*n*=12) groups. Water sample size: *n*=2 (repeated measures). Sample size varied according to the availability of animals at the time. Correlation analyses were performed using the life-stage data to examine the relationships between hepatic and plasma bile salt concentrations from the same animals.

## RESULTS

### Life-stage and sex differences in bile salt production

**Fig. 2.**
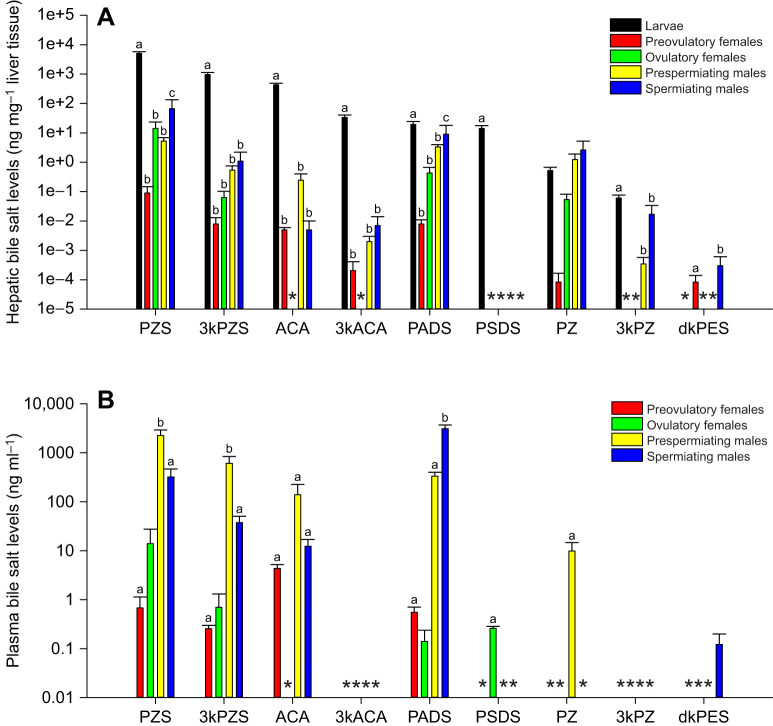
**Life-stage and sex differences in bile salt levels in sea lampreys.** (A) Hepatic bile salt levels. (B) Plasma bile salt levels. Larval liver had higher amounts of bile salts than the liver of preovulatory and ovulatory females, and prespermiating and spermiating males (one-way ANOVA, d.f.=4, *P*<0.0001), except PZ (one-way ANOVA, d.f.=4, *P*=0.1673). Comparisons among preovulatory and ovulatory females, and prespermiating and spermiating males showed sex differences in PZS (one-way ANOVA, d.f.=3, *P*=0.0159) and PADS (one-way ANOVA, d.f.=3, *P*=0.0016). Spermiating males had higher amounts of hepatic PZS (Bonferroni/Dunn *post hoc* test, *P*=0.0038) and PADS (Bonferroni/Dunn *post hoc* test, *P*=0.0002) than preovulatory females. Plasma bile salt concentrations were different among preovulatory and ovulatory females, and prespermiating and spermiating males (one-way ANOVA, d.f.=3, *P*<0.005). Prespermiating males had the highest amounts of PZS in the plasma (one-way ANOVA, d.f.=3, *P*=0.0001), significantly different from those in preovulatory females (Bonferroni/Dunn *post hoc* test, *P*<0.0001) and spermiating males (Bonferroni/Dunn *post hoc* test, *P*=0.0001). Prespermiating males also had the highest amounts of 3kPZS in the plasma (one-way ANOVA, d.f.=3, *P*=0.0015), significantly different from those in preovulatory females (Bonferroni/Dunn *post hoc* test, *P*=0.0008) and spermiating males (Bonferroni/Dunn *post hoc* test, *P*=0.0005). Spermiating males had the highest amounts of PADS in the plasma (one-way ANOVA, d.f.=3, *P*<0.0001), significantly different from those in preovulatory females (Bonferroni/Dunn *post hoc* test, *P*<0.0001) and prespermiating males (Bonferroni/Dunn *post hoc* test, *P*<0.0001). Data are presented as means±s.e.m. Different lowercase letters indicate values that are statistically different. *Non-detectable. Limit of detection for lamprey bile salts ranged from 0.009 ng ml^−1^ to 0.02 ng ml^−1^ in plasma and tissues, and 0.4 pg ml^−1^ to 0.05 ng ml^−1^ in water (Li et al., 2011; Wang et al., 2013, 2015). Note that the *y*-axis is a log scale. Liver sample size: larvae, *n*=21; preovulatory females, *n*=24; ovulatory females, *n*=5; prespermiating males, *n*=23; spermiating males, *n*=23. Plasma sample size: preovulatory females, *n*=40; ovulatory females, *n*=5; prespermiating males, *n*=43; spermiating males, *n*=59.

To investigate whether bile salt production is life-stage and sex dependent in sea lampreys, we measured bile salt concentration in liver tissue of larvae, preovulatory and ovulatory females, and prespermiating and spermiating males. Sea lampreys lose their gall bladder during metamorphosis (Youson and Sidon, 1978); therefore, larval liver still contains an intact gall bladder while adult liver does not. In general, larval liver had higher amounts of bile salts than the liver of preovulatory and ovulatory females, or prespermiating and spermiating males (one-way ANOVA, d.f.=4, *P*<0.0001), except PZ (one-way ANOVA, d.f.=4, *P*=0.1673), likely as a result of the accumulation of bile salts in the gall bladder (Fig. 2A). Interestingly, larval liver did not have detectable dkPES (Fig. 2A).

To discern the sex differences in bile salt production, we also compared hepatic bile salt concentrations among preovulatory and ovulatory females, and prespermiating and spermiating males. Only PZS (one-way ANOVA, d.f.=3, *P*=0.0159) and PADS (one-way ANOVA, d.f.=3, *P*=0.0016) showed significant sex differences. Spermiating males had higher amounts of hepatic PZS (Bonferroni/Dunn *post hoc* test, *P*=0.0038) and PADS (Bonferroni/Dunn *post hoc* test, *P*=0.0002) than preovulatory females (Fig. 2A). Notably, ovulatory females only produced detectable amounts of PZS, 3kPZS, PADS and PZ in the liver (Fig. 2A). None of the adults had detectable amounts of PSDS in the liver. In addition, preovulatory females did not have detectable amounts of hepatic 3kPZ, and prespermiating males did not have detectable amounts of hepatic dkPES (Fig. 2A).

Plasma bile salt concentrations were significantly different among preovulatory and ovulatory females, and prespermiating and spermiating males (Fig. 2B; one-way ANOVA, d.f.=3, *P*<0.005). Prespermiating males had the highest amounts of plasma PZS (one-way ANOVA, d.f.=3, *P*=0.0001), significantly different from those in preovulatory females (Bonferroni/Dunn *post hoc* test, *P*<0.0001) and spermiating males (Bonferroni/Dunn *post hoc* test, *P*=0.0001). Prespermiating males also had the highest amounts of plasma 3kPZS (one-way ANOVA, d.f.=3, *P*=0.0015), significantly different from those in preovulatory females (Bonferroni/Dunn *post hoc* test, *P*=0.0008) and spermiating males (Bonferroni/Dunn *post hoc* test, *P*=0.0005). Spermiating males had the highest amounts of plasma PADS (one-way ANOVA, d.f.=3, *P*<0.0001), significantly different from those in preovulatory females (Bonferroni/Dunn *post hoc* test, *P*<0.0001) and prespermiating males (Bonferroni/Dunn *post hoc* test, *P*<0.0001). None of the adults had detectable amounts of plasma 3kACA and 3kPZ, and ovulatory females did not have detectable amounts of plasma ACA. Plasma PSDS was only detected in ovulatory females, plasma PZ was only detected in prespermiating males, and plasma dkPES was only detected in spermiating males (Fig. 2B).

Taken together, 3kPZ and 3kACA were likely produced and sequestered in the tissues and not released into the circulation. Interestingly, PSDS was only detected in larval liver, but was present in the plasma of ovulatory females, indicating an extra-hepatic site for PSDS biosynthesis in ovulatory females. However, further investigation is required to confirm this speculation.

To determine the relationships between hepatic and plasma bile salt concentrations, correlation analyses were performed. As expected, hepatic bile salt concentrations were positively correlated with their plasma concentrations, and bile salts in the same branch of the synthetic pathway were positively correlated with each other (Table 1, *P*<0.05). Interestingly, plasma dkPES concentration was also positively correlated with hepatic PZS and PADS concentrations (Table 1, *P*<0.05).

**Table 1.**
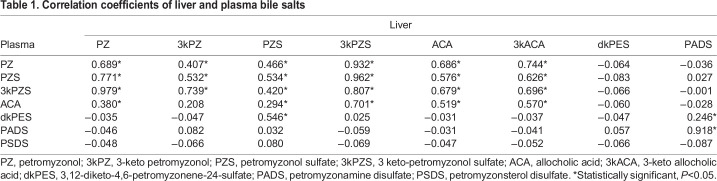
Correlation coefficients of liver and plasma bile salts

### Waterborne pheromones differentially alter bile salt production and clearance in preovulatory female sea lampreys

**Fig. 3.**
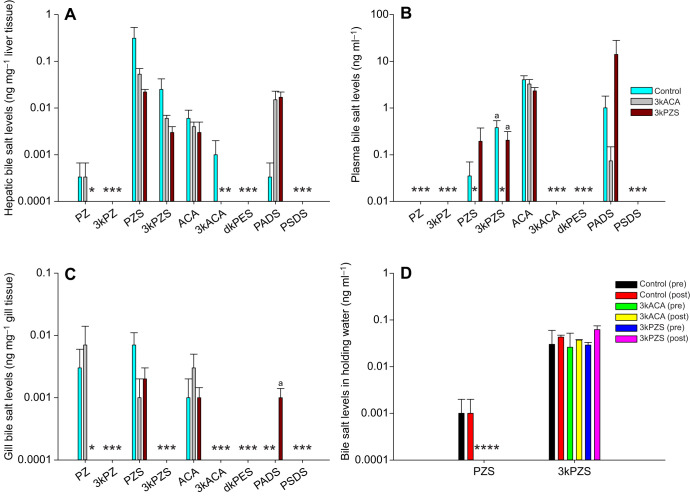
**Waterborne pheromones exert differential effects on bile salt production, circulation and clearance in preovulatory female sea lampreys.** (A) Hepatic bile salt levels, (B) plasma bile salt levels, (C) gill bile salt levels and (D) bile salt levels in holding water. Preovulatory female sea lampreys were exposed to waterborne vehicle (0.05 ppm methanol), 10^−10^ mol l^−1^ 3kACA or 10^−10^ mol l^−1^ 3kPZS for 4 h. Water samples (10 ml) were collected from the holding tank immediately before and after treatment (repeated measures). Exposure to 3kACA led to a reduction of hepatic 3kACA, and PZS and 3kPZS (one-way ANOVA, *P*=0.0167). Exposure to 3kPZS led to a reduction of hepatic PZ and 3kACA, and gill PZ, but increased gill PADS (one-way ANOVA, *P*=0.0332). Data are presented as means±s.e.m. Lowercase ‘a’ indicates values that are not statistically different. *Non-detectable. Limit of detection for lamprey bile salts ranged from 0.009 ng ml^−1^ to 0.02 ng ml^−1^ in plasma and tissues, and 0.4 pg ml^−1^ to 0.05 ng ml^−1^ in water (Li et al., 2011; Wang et al., 2013, 2015). Note that the *y*-axis is a log scale. Liver sample size: *n*=6 per group. Plasma sample size: control, *n*=8; 3kACA treated, *n*=8; and 3kPZS treated, *n*=10. Gill sample size: *n*=6 per group. Water sample size: *n*=2 (repeated measures). Sample size varied according to the availability of animals at the time.

Exposure to waterborne 3kACA led to a reduction of hepatic 3kACA (Fig. 3A) and plasma PZS and 3kPZS (one-way ANOVA, *P*=0.0167; Fig. 3B) in preovulatory females. However, exposure to waterborne 3kPZS led to a reduction of hepatic PZ and 3kACA (Fig. 3A), and gill PZ (Fig. 3C), but an increase of gill PADS (one-way test, *P*=0.0332; Fig. 3C) in preovulatory females. It seems that preovulatory females did not produce 3kPZ or PSDS. PZ was produced and sequestered in the liver and gill, and 3kACA was produced and sequestered in the liver locally because neither of them was circulating in the blood. In addition, preovulatory females only released trace amounts of PZS and 3kPZS into the water, and pheromone exposure did not affect bile salt release into the water (Fig. 3D). The release of 3kPZS seemed to be continuous because it was detectable in all groups before and after treatment. However, the release of PZS was not continuous as it was not detected in some treatment groups even before treatment. Interestingly, waterborne 3kACA seemed to inhibit the production of 3kACA in the liver and facilitate the clearance of PZS and 3kPZS in the plasma. In contrast, waterborne 3kPZS seemed to inhibit the production of C_24_ bile salts (PZ and/or 3kACA) in the liver and gill but facilitate the production of C_27_ bile salt PADS in the gill.

### Waterborne pheromones differentially alter bile salt production in prespermiating male sea lampreys

**Fig. 4.**
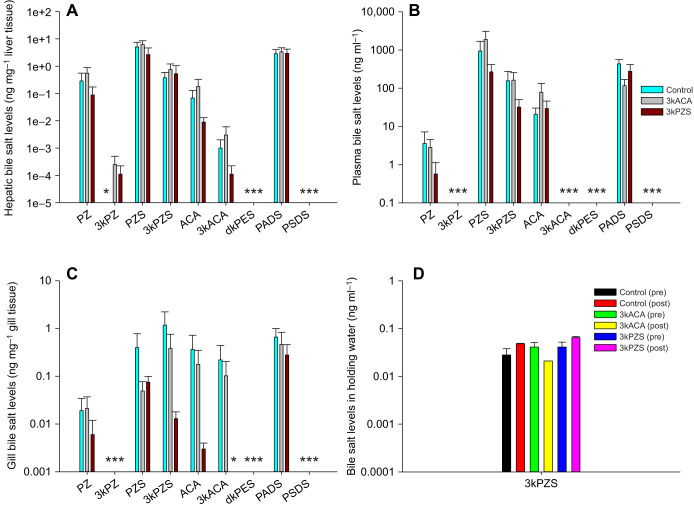
**Waterborne pheromones exert differential effects on bile salt production, circulation and clearance in prespermiating male sea lampreys.** (A) Hepatic bile salt levels, (B) plasma bile salt levels, (C) gill bile salt levels and (D) bile salt levels in holding water. Prespermiating male sea lampreys were exposed to waterborne vehicle (0.05 ppm methanol), 10^−10^ mol l^−1^ 3kACA or 10^−10^ mol l^−1^ 3kPZS for 4 h. Water samples (10 ml) were collected from the holding tank immediately before and after treatment (repeated measures). Exposure to 3kACA increased hepatic 3kPZ while exposure to 3kPZS increased hepatic 3kPZ but led to a reduction of gill 3kACA. Data are presented as means±s.e.m. *Non-detectable. Limit of detection for lamprey bile salts ranged from 0.009 ng ml^−1^ to 0.02 ng ml^−1^ in plasma and tissues, and 0.4 pg ml^−1^ to 0.05 ng ml^−1^ in water (Li et al., 2011; Wang et al., 2013, 2015). Note that the *y*-axis is a log scale. Liver sample size: control, *n*=11; 3kACA treated, *n*=12; and 3kPZS treated, *n*=9. Plasma sample size: control, *n*=13; 3kACA treated, *n*=12; and 3kPZS treated, *n*=7. Gill sample size: *n*=6 per group except 3kPZS treated group (*n*=3). Water sample size: *n*=2 (repeated measures). Sample size varied according to the availability of animals at the time.

Exposure to waterborne 3kACA increased hepatic 3kPZ (Fig. 4A), whereas exposure to waterborne 3kPZS increased hepatic 3kPZ (Fig. 4A) but led to a reduction of gill 3kACA (Fig. 4C) in prespermiating males. It seems that prespermiating males did not produce dkPES and PSDS (Fig. 4). 3kPZ was only produced and sequestered in the liver (Fig. 4A), and 3kACA was produced and sequestered in the liver and gill because neither of them was circulating in the blood (Fig. 4B). Both pheromones seemed to induce 3kPZ production in the liver but have no effect on plasma bile salt levels. In addition, prespermiating males only released trace amounts of 3kPZS into the water, and pheromone exposure did not alter bile salt release into the water (Fig. 4D).

### Waterborne pheromones differentially alter bile salt distribution and 3kPZS increases bile salt release into water in spermiating male sea lampreys

**Fig. 5.**
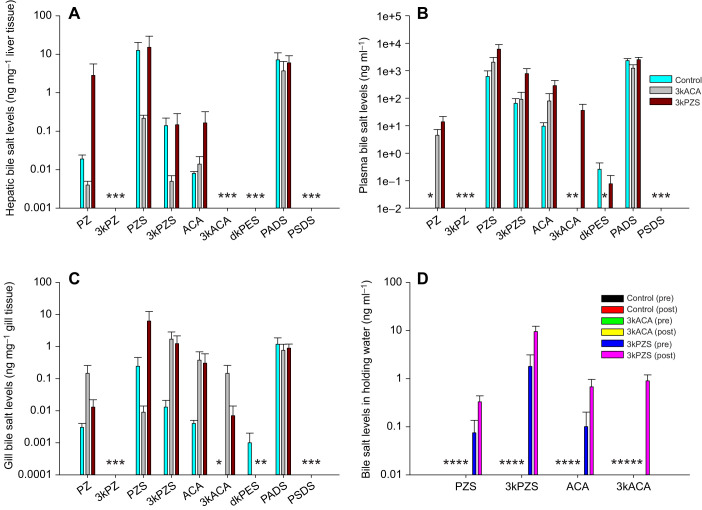
**Waterborne pheromones exert differential effects on bile salt production, circulation, clearance and release in spermiating male sea lampreys.** (A) Hepatic bile salt levels, (B) plasma bile salt levels, (C) gill bile salt levels and (D) bile salt levels in holding water. Prespermiating male sea lampreys were exposed to waterborne vehicle (0.05 ppm methanol), 10^−10^ mol l^−1^ 3kACA or 10^−10^ mol l^−1^ 3kPZS for 4 h. However, these animals began spermiating at the time of sample collection. Water samples (10 ml) were collected from the holding tank immediately before and after treatment (repeated measures). Pheromone exposure had no significant effect on hepatic bile salt levels. Exposure to 3kACA increased PZ, but led to the clearance of dkPES in the plasma, and increased gill 3kACA. Exposure to 3kPZS increased plasma PZ, and plasma and gill 3kACA. 3kPZS also increased the release of PZS, 3kPZS, ACA and 3kACA into water, whereas 3kACA had no effect on the release of bile salts into water. *Non-detectable. Limit of detection for lamprey bile salts ranged from 0.009 ng ml^−1^ to 0.02 ng ml^−1^ in plasma and tissues, and 0.4 pg ml^−1^ to 0.05 ng ml^−1^ in water (Li et al., 2011; Wang et al., 2013, 2015). Note that the *y*-axis is a log scale. Liver sample size: *n*=6 per group except the 3kACA-treated group (*n*=3). Plasma sample size: control, *n*=23; 3kACA treated, *n*=16; and 3kPZS treated, *n*=26. Gill sample size: control, *n*=11; 3kACA treated, *n*=9; and 3kPZS treated, *n*=12. Water sample size: *n*=2 (repeated measures). Sample size varied according to the availability of animals at the time.

In spermiating males, pheromone exposure had no significant effects on hepatic bile salt levels (Fig. 5A). Exposure to waterborne 3kACA increased PZ but led to the clearance of dkPES in the plasma (Fig. 5B), and increased gill 3kACA in spermiating males. Exposure to waterborne 3kPZS increased plasma PZ (Fig. 5B), as well as plasma and gill 3kACA (Figs 5B,C) in spermiating males. In addition, exposure to waterborne 3kPZS increased the release of PZS, 3kPZS, ACA and 3kACA into the water, whereas exposure to waterborne 3kACA had no effect on bile salt release into the water (Fig. 5D). It seems that spermiating males did not produce PSDS. Pheromone exposure seemed to favor the clearance of dkPES while also facilitating the transportation of PZ and 3kACA in the plasma, and 3kACA uptake/production and water release in the gill.

## DISCUSSION

We found that sea lampreys produced distinct mixtures of bile salts at different life stages. It is likely that different bile acid synthetic enzymes were upregulated or downregulated at specific life stages. For example, sea lamprey larvae did not produce detectable amounts of dkPES, probably because of the lack of 3β-hydroxy-Δ^5^-C_27_-steroid dehydrogenase/isomerase (HSD3B7) activity (Chiang, 2004). In contrast, most adults (except ovulatory females) did not produce detectable amounts of PSDS, possibly as a result of the repression of a C3-specific sulfotransferase. Via the up- or down-regulation of different hydroxylases, aldo-keto reductases and C24-specific sulfotransferase (Chiang, 2004; Venkatachalam et al., 2004), these bile acid synthetic enzymes potentially play important roles in producing distinct mixtures of bile salts at different life stages. However, further investigation is required to confirm this speculation.

We found life-stage and sex differences in bile salt levels in sea lampreys. This phenomenon persists in the spawning stage where only spermiating males release a large quantity of bile salts as sex pheromones, whereas ovulatory females barely release any detectable amounts of bile salts (Brant et al., 2013; Li et al., 2002, 2017b). Evidently, pheromonal bile salts exert many sex-dependent responses (Brant et al., 2016; Buchinger et al., 2013, 2017; Johnson et al., 2006, 2009; Siefkes et al., 2005; Walaszczyk et al., 2013, 2016). For example, 3kPZS evokes context-dependent changes in brain serotonin levels in preovulatory females, but not in prespermiating males (Chung-Davidson et al., 2015). However, 3kPZS increases gonadotropin-releasing hormone and steroidal outputs in prespermiating males, but less so in preovulatory females (Chung-Davidson et al., 2013a). Interestingly, 3kPZS and 3kACA exert differential effects on gonadotropin-inhibitory hormone (GnIH)-related neuropeptide levels in prespawning adults (Chung-Davidson et al., 2020b), and GnIH increases the expression of 3β-hydroxysteroid dehydrogenase/Δ5–Δ4 isomerase, a steroidogenic enzyme (Qi et al., 2013; Wang et al., 2017). The differential effects of pheromones on bile salt production could be mediated through GnIH-related mechanisms. However, further investigation is required to uncover the underlying mechanisms.

We found that pheromonal bile salts exerted differential effects on bile salt biosynthesis, circulation and clearance. In addition, 3kPZS only induced bile salt release in spermiating males. PZS was the first bile salt identified in sea lamprey larvae (Haslewood and Tökes, 1969), and it seems to be the most abundant bile salt in larvae and prespawning adults based on the results in this study. The biosynthetic pathway of PZS is considered unique to lamprey species, and its precursor PZ can be converted from ACA (Haslewood and Tökes, 1969). Judging by the variations in structural modifications of known bile salts, lamprey bile acid synthetic pathways appeared less restricted than those in mammals (Chiang, 2004; Elliott and Hyde, 1971; Russell and Setchell, 1992; Salen and Shefer, 1983). For instance, the modifications at C3, C5, C7, C12 and C24 positions seemed to be fluid, suggesting that bile salt synthetic enzymes in lampreys may be more promiscuous in substrate selection, and less compartmentalized as in mammals (Chiang, 2004; Hagey et al., 2010; Midzak and Papadopoulos, 2014; Suld et al., 1962).

Bile acid synthesis is regulated by multiple feedback inhibition mechanisms in mammals (Chiang, 2004). One mechanism involves bile acid chenodeoxycholate and cytokine IL-1β. They inhibit HNF4α but induce c-Jun, which in turn blocks HNF4α recruitment of PGC-1α to cholesterol 7α-hydroxylase (CYP7A1, rate limiting enzyme for bile acid biosynthesis) chromatin. This results in the inhibition of *CYP7A1* gene expression. Therefore, the JNK/c-Jun signaling pathway inhibits bile acid synthesis and protects hepatocytes against the toxic effect of inflammatory agents (Li et al., 2006). As 3kACA and 3kPZS differentially alter JNK/c-Jun expression in prespawning sea lampreys (Chung-Davidson et al., 2013a,b), it is likely that they may act through similar JNK/c-Jun inhibitory mechanisms.

One interesting finding in this study is that not all bile salts were produced continuously in the liver, and not all bile salts were released into the circulation and water. In prespawning sea lampreys, 3kACA and 3kPZ are more likely metabolic intermediates produced in and confined to the liver, whereas other bile salts are more likely produced in the liver and exert remote functions via the circulation. In spermiating males, 3kPZS was the major pheromonal bile salt released into the water accompanied by lesser amounts of PZS, ACA and 3kACA, and the release could be influenced by exogenous 3kPZS. Interestingly, sea lampreys contain other organs that can produce, modify or release bile salts, such as the gill and intestine (Li et al., 2002; Siefkes et al., 2003; Yeh et al., 2012). Therefore, bile salts in sea lamprey may exhibit autocrine and endocrine functions.

In summary, bile salt levels are life-stage and sex dependent in sea lampreys. Pheromonal bile salts exert differential effects on bile salt production, transportation and clearance in prespawning adults, whereas only 3kPZS induces the water release of bile salt in spermiating males.
